# Supplementary material for: Complexome profiling of the Chlamydomonas psb28 mutant reveals TEF5 as an early PSII assembly factor
Source: Plant Cell. 2025 Mar 17;37(6):koaf055. doi: 10.1093/plcell/koaf055 (PMC12164590; doi:10.1093/plcell/koaf055)
Supplement: koaf055_Supplementary_Data [file koaf055_supplementary_data.zip › psb28_interactive.zip!/plots/intensity/qualityPlots/peptides/QCR9_Cre11.g468950.t1.2.html]

### Cre11.g468950.t1.2

Trivial name: QCR9  
SeqCoverage [median]: 78.30  
Mass: 7.03 kDa  
  
 

| Sequence | MissedCleavage | Mass [Da] | wtIntensity | muIntensity | Score |
| --- | --- | --- | --- | --- | --- |
| LFSDIIAER | 0 | 1062.57 | 21903.34 | 33647.23 | 124.23 |
| LFSDIIAERT | 1 | 1163.62 | 69130.87 | 113956.91 | 154.56 |
| LSEALYQTFFK | 0 | 1345.69 | 21029.74 | 7510.53 | 247.54 |
| LSEALYQTFFKR | 1 | 1501.79 | 12697.78 | 18979.46 | 148.28 |
| STVYIPMLLVGAYFSNEAIDYAVDK | 0 | 2778.38 | 535.47 | 0.00 | 65.42 |
|  |  |  | 125297.2 | 174094.1 |  |
